# Addressing COVID-19 in Malawi

**DOI:** 10.11604/pamj.supp.2020.35.2.23960

**Published:** 2020-06-09

**Authors:** Parth Patel, Yusuff Adebayo Adebisi, Munharo Steven, Don Eliseo Lucero-Prisno III

**Affiliations:** 1 Department of Health Systems and Policy, College of Medicine, University of Malawi; 2 Faculty of Pharmacy, University of Ibadan, Ibadan, Nigeria; 3 Training and Research Unit of Excellence, College of Medicine, University of Malawi; 4 Department of Global Health and Development, London School of Hygiene and Tropical Medicine, United Kingdom

**Keywords:** 2019-nCoV, SARS-CoV-2, COVID-19, coronavirus, Malawi, Africa, outbreak, global health, pandemic

## Abstract

**Introduction**: COVID-19 is a global public health threat that Africa faces including the country Malawi. With an already burdened health system and an economically challenged population due to the poverty level, Malawi is suddenly faced by a pandemic that will test the country’s healthcare systems. Its government has already instituted an array of initiatives and plans and funding efforts toward the effective containment of the pandemic. Some of these efforts include reorientation and training for health workers, securing funding to procure and distribute needed personal protective equipment, medicine supply, health promotion, surveillance and case management. These efforts are being done in the context of an already heavy burden of diseases such as HIV/AIDS, malnutrition and poor health literacy. Whether efforts of the government and other stakeholders are enough, these remain to be seen in containing the virus and its aftermath. Until an effective treatment or vaccine is available, Malawi has to remain vigilant and needs to continue all its efforts to address its COVID-19 epidemic.

## Commentary

COVID-19 presents major challenges to Africa as the virus finds its way to the inroads of the continent [1-3] including the country of Malawi. This sub-Saharan African country, which is already experiencing political and economic difficulties, has its weak health systems suddenly facing the formidable task of quelling the virus. After the first 3 cases were identified on 2 April 2020 in Lilongwe among returning Malawians from abroad, this landlocked country with a population of over 18 million and a population density of 129 people per square kilometer (ranking among the top 10 countries in the region by density) is suddenly faced by a formidable challenge. As of 10 May 2020, Malawi had 57 confirmed cases with 3 deaths and by 30 May 2020, the country had 273 cases and 4 deaths [4]. This contributes to the uptrending mortality rate and incidence rate of Africa where 7 countries have reported fatality rates higher than the global case fatality rate of 6.9% [3]. In this commentary, we provide a quick analysis of the current state of the COVID-19 pandemic in Malawi and its efforts to address it.

While the rest of the world was battling their own epidemics before the first cases were reported in Malawi, the government already made the pronouncements that the pandemic is a national threat. Early measures were put in place including the banning of public gatherings of more than a hundred people in places such as churches, mosques, meetings, rallies, conferences, weddings and funerals. When the situation necessitated implementing a lockdown strategy, the government suddenly faced a major impediment when a legal action was undertaken by the Human Rights Defenders Coalition in the High Court of Malawi which resulted in an injunction that blocked government´s attempts to execute the measure [5]. This is one of the unique global instances where a legal argument prevailed over public health. The Malawian health sector which battles the epidemic is already challenged by inadequate funding, insufficient staffing, battered infrastructure, and lack of essential medicines and equipment. Together with a high poverty level and poor health literacy, all these posed further challenges towards an effective containment. Aggravating the already tense situation, health personnel went on strike for two weeks forcing the government to employ more personnel, provide enough personal protective equipment and increase hazard allowances. Inadequacy of equipment adds to the grim picture as the country has a total of only 25 intensive care units (ICU) and 7 functioning ventilators [6]. This would render frontline health personnel incapable of mounting an adequate response to critically ill COVID-19 patients who would require ventilation in the event of need during the possible surge of patients during peaks of epidemics as experienced in many countries [7]. When it comes to testing capacity, as of 30 May 2020, Malawi had tested not more than 4,320 suspected cases [4]. This is already a modest achievement on the part of the Malawian government which has also equipped some districts in the country such as Mzimba District Hospital and Mzuzu Central Hospital with the capacity to test COVID-19 apart from already existing facilities such as College of Medicine, Malawi Liverpool Wellcome Trust and the National Public Health Reference Laboratory. Thus, it becomes imperative that the country continues to scale-up its testing capacity.

The government launched a masterplan, the National COVID-19 Preparedness and Response Plan, which came into effect in March and to finish in June 2020 to help guide in the protection of its citizens by reducing the risk of exposure to the virus. It also kept on working with the Africa Centre for Disease Control and WHO African Region. This plan includes: reorientation and training of personnel who work in highly infectious environments, securing funding to procure and distribute needed personal protective equipment, medicine supply, investigation, and case management. Screening and surveillance at borders and all points of entry, enforcing quarantine or recommending self-quarantine (due to insufficient quarantine facilities) for 14 days for all people coming from COVID-19 high-risk countries were also intensified as part of this plan [8]. Health education and information sharing were ramped up at the community level through distribution of posters and leaflets, and broadcasting jingles. The Ministry of Health created awareness and promoted preventive measures including hand and respiratory hygiene and physical distancing while the Ministry of Science, Education and Technology suspended classes indefinitely in schools and institutions of higher learning. The government developed an application to track COVID-19 patients and to ensure compliance with the guidelines. Information on COVID-19 is accessible through a national toll-free line and through social media. The Malawi Prison Services and Juvenile Centres released prisoners with minor offences and those who have served moderate period of their punishment in prison to decongest the country’s prisons in the effort to reduce the spread of the virus. These prisoners were presented to the Minister of Homeland Security for monitoring.

The need to fund the COVID-19 fight was recognized by the government by providing a budget of $213 million to the National COVID-19 Preparedness and Response Plan. In preparation of augmenting this budget, discussions have already been initiated with the International Monetary Fund for a budget of $150 million. A National Relief Fund to accept contributions from donors, private companies and individuals was also set up. The response plan involves $20 million spending on health care efforts and hiring 2000 extra health personnel. Efforts have been implemented to shield vulnerable populations from socio-economic impacts of the pandemic through the provision of $40 monthly payments that is equivalent to the country’s minimum wage. This benefits one million people and small business personnel [9]. The United Nations in Malawi and the World Bank launched an emergency appeal fund of $140 million and $37 million respectively to help the country cope with COVID-19 preparedness and response which included detecting and confirming cases, equipping facilities and treating patients.

These efforts of the country to curb the spread of COVID-19 and mitigate its impact may be commendable, however the challenges remain colossal. The country´s health system is already burdened with HIV/AIDS with a prevalence among adults at 9.2%, of which only 69% are virally suppressed [8]. This will render the noncompliant patients susceptible to comorbid infections, most commonly TB, pneumonia, meningitis. Malnutrition is also one of the major public health issues facing African countries [10] including Malawi. Malnutrition is rampant with about 42.4% of the under 5 population having severe acute malnutrition. This situation gradually progresses into adulthood with little to no improvements in nutritional status, further worsening the immunity of the individuals who had it before. These extrinsic factors can adversely affect immune responses of individuals, producing states of secondary immunodeficiency and consequently predisposing them to infection with COVID-19.

## Conclusion

It is believed that many countries in Africa including Malawi are yet to feel the full impact of the pandemic. The current actions of the government are hinged on this premise thus the various programs and initiatives that it mounted. These preparations, if indeed enough, remain to be seen as its health system has already been facing many challenges prior to the pandemic. On the bright side, the country may capitalize on the seeming resilience of its health system which survived the ravages of historical diseases amongst its population. COVID-19 provides an extra jolt for the country to revisit its investment in health. There is no doubt that the health sector needs to be revitalized by creating a robust health environment for better health outcomes. Until an effective treatment or vaccine is available, the government of Malawi and other stakeholders need to continually strengthen its efforts to curb its COVID-19 epidemic. Who knows, there is a good spillover after this battle.

## Competing interests

The authors declare no competing interests.
